# Utility of Acute Physiology and Chronic Health Evaluation (APACHE II) in Predicting Mortality in Patients with Pyogenic Liver Abscess: A Retrospective Study

**DOI:** 10.3390/jcm10122644

**Published:** 2021-06-16

**Authors:** Yuan-Ti Lee, Chi-Chih Wang, Chien-Feng Li, Hsuan-Yi Chen, Hsien-Hua Liao, Chia-Chun Lin

**Affiliations:** 1 School of Medicine, Chung Shan Medical University, Taichung 40201, Taiwan; bananaudwang@gmail.com (C.-C.W.); jojo33033@gmail.com (C.-F.L.); circlerank@gmail.com (H.-Y.C.); lhhy011@gmail.com (H.-H.L.); 2 Division of Infectious Diseases, Department of Internal Medicine, Chung Shan Medical University Hospital, Taichung 40201, Taiwan; g9039029@gmail.com; 3 Division of Gastroenterology and Hepatology, Department of Internal Medicine, Chung Shan Medical University Hospital, Taichung 40201, Taiwan; 4 Department of Surgery and Intensive Care Units, Chung Shan Medical University Hospital, Taichung 40201, Taiwan

**Keywords:** acute physiology and chronic health evaluation II score, intensive care unit, klebsiella pneumoniae, mortality, pyogenic liver abscess, risk factors, sequential organ failure assessment

## Abstract

Pyogenic liver abscess (PLA) is a major life-threatening disease with varied clinical features. This study aimed to determine predictors of mortality in patients with PLA using criteria determined upon admission. We retrospectively examined the data of 324 hospitalized adults in whom liver abscesses were confirmed using abdominal ultrasound and/or computed tomography. The relationship between various risk factors was assessed using multivariate analysis. A total of 109 (33.6%) patients were admitted to the intensive care unit (ICU). The overall mortality rate was 7.4% and was higher among ICU patients than non-ICU patients (21.1% vs. 0.5%, *p* < 0.001). PLA patients with an Acute Physiology and Chronic Health Evaluation (APACHE) II score ≥18 had a 19.31-fold increased risk, and those with concomitant infections had a 34.33-fold increased risk of 30-day mortality according to multivariate analysis. The estimated area under the receiver operating characteristic curve for predicting 30-day mortality revealed that APACHE II score ≥18 (sensitivity of 75% and specificity of 84%, *p* < 0.0001) had better discriminative power than Sequential Organ Failure Assessment (SOFA) ≥6 (sensitivity of 81% and specificity of 66%, *p* < 0.0001). APACHE II has shown better discrimination ability than SOFA in predicting mortality in PLA patients. To improve outcomes in patients with PLA, future management strategies should focus on high-risk patients.

## 1. Introduction

The incidence of pyogenic liver abscess (PLA), a major life-threatening disease with varied clinical features, is increasing worldwide. Currently, the epidemiology of PLA is geographically diverse, depending on the population prevalence, age, sex, and acquisition mode [1,2,3,4,5,6,7,8]. Taiwan is considered to have a high incidence of PLA (15.45 per 100,000 people) [3,9]. Additionally, the incidence of PLA is greater in patients with diabetes than in those without diabetes (115.40 per 100,000 people and 36.58 per 100,000 people, respectively) [10]. Despite continuous advancements in medical technology enabling physicians to better diagnose and treat patients with PLA [11], the mortality rate due to PLA varies from 2% to 28% [2,3,4,5,12,13,14,15,16,17]. This is of particular concern in critically ill patients admitted to the intensive care unit (ICU) as they have more adverse outcomes and a high risk of mortality [3,6,16].

Factors associated with increased mortality due to PLA include the following: age ≥65 years; male sex; presence of diabetes, malignancy, and liver or/and biliary disease; *Klebsiella pneumoniae* (*K. pneumoniae*) infections; multidrug resistance; polymicrobial or mixed anaerobic infections; gas-forming abscesses; abscess ruptures; multiloculated abscesses; metastatic infections; inappropriate initial antibiotics; Acute Physiology and Chronic Health Evaluation (APACHE) II scores ≥15; ICU stay; septic shock; respiratory failure with mechanical ventilation; and multiple organ failures [1,2,3,6,13,15,17,18,19].

APACHE II and Sequential Organ Failure Assessment (SOFA) scores have been widely used to predict multiple organ failure and hospital mortality in ICU patients with sepsis or other infectious diseases [20,21,22,23]. Studies of current treatment strategies for critically ill patients with PLA have highlighted the nonspecific nature of these strategies; however, these studies have not assessed the risk factors associated with treatment outcomes in ICU patients with PLA [5,6,14,24]. Further, although the benefits of various mortality predictors have been shown to improve outcomes [2,14,17], the impact of risk factors on treatment and health outcomes in ICU patients remains unclear. Therefore, we collected the clinical characteristics, treatments, and outcomes in patients with PLA and determined the prognostic factors on admission for mortality within the first 30 days.

## 2. Materials and Methods

### 2.1. Study Design and Patient Selection

A retrospective review of patients admitted to the Chung Shan Medical University Hospital from 2013 to 2018 was performed by searching the hospital electronic medical records for patients with liver abscesses (International Classification of Disease, Ninth Revision, Clinical Modification [ICD-9-CM], Diagnosis Code: 572.0 and ICD-10-CM Diagnosis Code K75.0). 

The inclusion criteria were as follows: confirmed liver abscess (≥1 space-occupying lesion in the liver) [1] found using ultrasound and/or computed tomography (CT) with contrast enhancement of the abdomen, adults aged ≥20 years with positive or negative blood and/or abscess culture results, and community-acquired infections. The exclusion criteria were as follows: fungal or amoebic liver abscess, infected liver cyst, and those with missing data. In total, 338 patients with liver abscesses were included in the analysis. Patients were stratified into two groups: the ICU group (those who were admitted to the ICU) and non-ICU group (those who were admitted to the general ward). ICU admission, discharge, and triage guidelines followed those previously published [25]. 

### 2.2. Data Collection and Definition of the Variables

Data on demographics (sex and age), duration and unit of hospitalization, clinical manifestations, laboratory data and imaging at admission, comorbidities (diabetes mellitus, hypertension, biliary disease, chronic liver diseases (including hepatitis B and C), chronic kidney diseases, peptic ulcer, concomitant neoplasms, and alcohol consumption), and the results of antibiotic susceptibility testing for isolated pathogens were collected. 

Routine blood examinations included the following: complete blood count, serum biochemical tests (including fasting blood sugar, hemoglobin A1c, and liver and renal function), and high-sensitivity C-reactive protein. Consciousness level was evaluated using the Glasgow Coma Scale and severity of illness was estimated using the SOFA and APACHE II scores on admission [26,27,28]. The definition of sepsis and septic shock followed the International Sepsis Definitions Conference criteria [29].

Fever was defined as an ear temperature ≥37.8 °C. Multidrug-resistant organisms were defined as pathogens resistant to at least one agent in three or more antimicrobial categories on susceptibility testing [30]. Metastatic infection was defined as new extra-hepatobiliary infection sites with the same pathogen(s) as that in the PLA [1]. Patients with a history of PLA for >6 months affected with the same pathogen(s) were considered relapsed cases. Concomitant infections were defined as septic shock and bacteremia, pneumonia, and/or urinary tract infections caused by the same pathogen(s).

### 2.3. Treatment and Outcomes

We collected data on the duration of inpatient intravenous antimicrobial therapy and outpatient oral antimicrobial therapy. Empirical antibiotic therapy was defined as therapy administered within 24 h of performing the culture, with subsequent therapy modified based on the microbiological culture and susceptibility results or clinical response after antibiotic administration for ≥48 h [17]. Appropriate antibiotic therapy meant at least one antimicrobial agent to which the causative pathogen displayed in vitro susceptibility was utilized [31]. Inappropriate therapy meant the absence of antimicrobial agents to which an organism is susceptible or to which the organism was resistant [31]. Treatment outcomes evaluated included appropriate antimicrobial therapy alone [31], antimicrobial therapy combined with percutaneous catheter drainage (PCD), and/or surgical treatment. The survival follow-up data collected from electronic medical records, in which the patients were alive ≥30 days after admission to the hospital, were defined as the clinical responses of treatment outcome. The definition of failure of PCD included death while abscess drains were in place or when surgical treatment was required [15]. The need for surgical treatment was defined as when the patients either deteriorated or showed no improvement in clinical symptoms or signs, developed persistent abscesses, or suffered an abscess rupture after initial treatment with PCD despite multiple drainage endeavors [15]. Response to treatment was evaluated in each patient by a series of follow-up abdominal ultrasounds or CT scans of the liver either during hospitalization and/or after discharge from the hospital [15]. The definition of non-response to therapy included inappropriate therapy, failure of PCD, surgical treatment, and 30-day mortality.

Mortality included deaths that occurred in-hospital or after discharge within 30-days of the initial hospitalization. Causes of mortality were either death as a direct consequence of PLA or its complications.

### 2.4. Statistical Analyses

All data were analyzed using the SPSS 22.0 Statistical Software Package (SPSS, Inc., Chicago, IL, USA). Descriptive analysis was used to compare differences in demographics, clinical manifestations, illness severity, laboratory factors, and treatment between ICU and non-ICU participants. Continuous variables are presented as means (standard deviations (SDs)), while categorical data are presented as numbers (*n*) and percentages (%).

Independent two-sample *t*-tests or Mann–Whitney U-tests were used to analyze continuous variables. Categorical variables were compared using either chi-square or Fisher exact tests. The relationships among demographic characteristics, clinical manifestations, illness severity, laboratory factors, outcomes, and ICU stay were assessed using univariate analysis. 

Multivariate logistic regression models in the forward selection mode were applied to significant factors from the univariate analysis. Adjusted odds ratios (aORs) and 95% confidence intervals (95% CIs) were estimated from the logistic regression model, with aOR estimated by controlling the covariates. Scoring models fitting the logistic regression were incorporated into the prediction model, and their predictive performance was measured from the area under the curve (AUC) of the receiver operating characteristic (ROC) analysis. We used ROC analysis to test the discriminative ability of the APACHE II and SOFA scores to detect mortality. Differences in survival were assessed with the Kaplan–Meier method. All statistical tests were two-sided and evaluated at a 0.05 significance level.

## 3. Results

**Figure 1:**
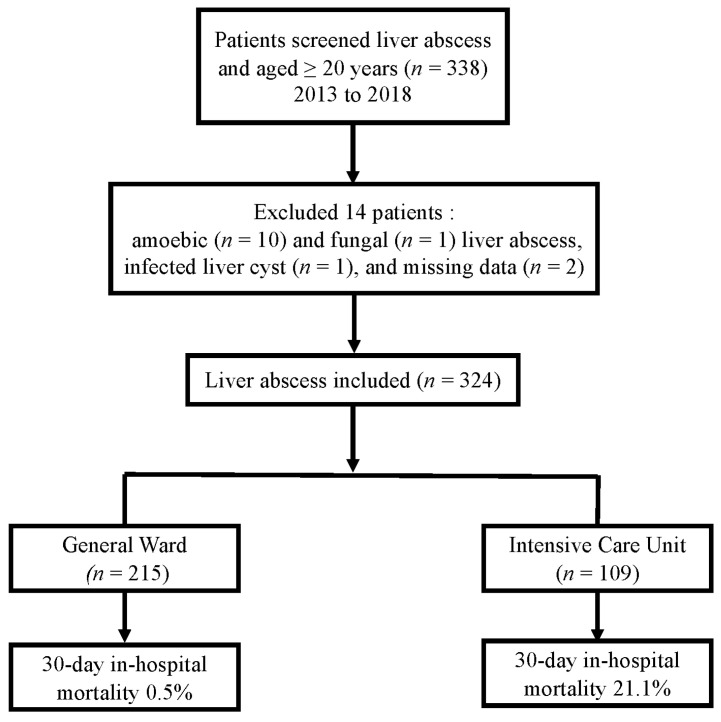
Flowchart of the included patients with liver abscesses and mortality.

After reviewing the medical records to identify patients with PLA, we excluded 14 patients based on the following criteria: presence of amoebic (*n* = 10) or fungal (*n* = 1) liver abscess, infected liver cyst (*n* = 1), or missing data (*n* = 2). Thus, 324 patients with PLA were eventually included in this study (Figure 1). Baseline characteristics of the patients are shown in Table 1. The results of treatment, use of antimicrobial agents, and treatment outcomes are shown in Table 2.

A total of 109 patients with PLA were admitted to the ICU during the study period. Patients in the ICU group were older (mean age, 62.9 years), had longer hospital stays (mean length of stay, 26.1 days), and had a higher prevalence of diabetes (39.1%) and hypertension (34.4%) than those in the non-ICU group. The illness severity of patients admitted to the ICU was significantly higher than that of non-ICU patients. ICU patients had higher SOFA (≥6, 68.8%) and APACHE II scores (score ≥18, 38.5%) than non-ICU patients (*p* < 0.001).

### 3.1. Clinical Manifestations and Laboratory, Microbiologic, and Imaging Findings

Patients with PLA in the general ward experienced fever (90.2 vs. 80.7%, *p* = 0.016), especially fever >38.3 °C (71.2 vs. 58.7%, *p* = 0.024) and chills (71.2 vs. 58.7%, *p* = 0.024); more details can be found in Table S1. Chest pain (19.3 vs. 10.2%, *p* = 0.024) and dyspnea (22 vs. 12.6%, *p* = 0.027) were more common in the ICU group than in the non-ICU group. On physical examination, the following signs were more common among ICU patients than among non-ICU patients: lower systolic blood pressure (108.1 ± 25.9 vs. 117.9 ± 18.6 mmHg, *p* = 0.001), mental confusion (18.3 vs. 2.8%, *p* < 0.001), abnormal breath sounds on auscultation (25.7 vs. 10.7%, *p* < 0.001), and ascites (16.5 vs. 6.0%, *p* = 0.002). The ICU patients also had a higher incidence of anemia (21.4 vs. 6.6%, *p* <0.001), thrombocytopenia (32.1 vs. 8.8%, *p* < 0.001), elevated aspartate aminotransferase (64 vs. 39.5%, *p* < 0.001), increased alkaline phosphatase (59.3 vs. 43.7%, *p* = 0.017), hyperbilirubinemia (56 vs. 43%, *p* = 0.039), elevated blood urea nitrogen (64.7 vs. 32.7%, *p* < 0.001), and increased creatinine (54.1 vs. 22.8%, *p* < 0.001) than the non-ICU patients. 

A total of 255 bacterial isolates were obtained from 324 patients (103 species from both blood and abscess samples, 78 from blood samples, and 74 from abscess samples); 69 patients had negative culture results; more details can be found in Table S2. Bacteremia was more common among ICU patients (32.1%) than among non-ICU patients. A total of 182 isolates of *K. pneumoniae* were extracted; *K. pneumoniae* bacteremia (51.4%) and multidrug-resistant organisms (26.6%) were more common in ICU patients than in non-ICU patients. In imaging studies, there were no differences in the size and number of abscesses between the two groups.

### 3.2. Treatment, Complications, and Outcomes

Compared with those in the non-ICU group, patients in the ICU group had a significantly longer duration of parenteral antibiotic therapy (mean duration, 21.7 days). More ICU patients required subsequent antibiotic treatment (55%) than non-ICU patients. A total of 182 patients (56.2%) received intravenous antibiotic treatment combined with PCD. Only 17 patients underwent surgical treatment; among them, more patients belonged to the ICU group (11/109) than the non-ICU group (6/215).

The ICU patients had a significantly higher no-response rate to antibiotics combined with PCD (26.6%) than the non-ICU patients (14.4%). The overall in-hospital mortality rate was 7.4%, and significantly more ICU patients died (21.1%, *p* < 0.001) than non-ICU patients (0.5%). Metastatic infections (8.3%, *p =* 0.032) and concomitant infections (60.6%, *p* < 0.001) were more common in ICU patients. 

### 3.3. Multivariate Analyses of Clinical Factors in Relation to 30-Day Mortality 

Given the higher fatality rate in the ICU group, we performed a logistic regression model to predict the risk factors associated with 30-day mortality related to PLA. Table 3 shows the factors derived from the univariate and multivariate analyses that were associated with the mortality of PLA patients. In multivariate analysis, the significant factors for predicting mortality included the following: high APACHE II score (aOR: 19.31, 95% CI: 4.77–78.22) and concomitant infections (aOR: 34.33, 95% CI: 5.60–210.55).

### 3.4. ROC and Kaplan–Meier Curve Analyses of APACHE II and SOFA Scores and Cutoff Points Predicting 30-Day Mortality

**Figure 2:**
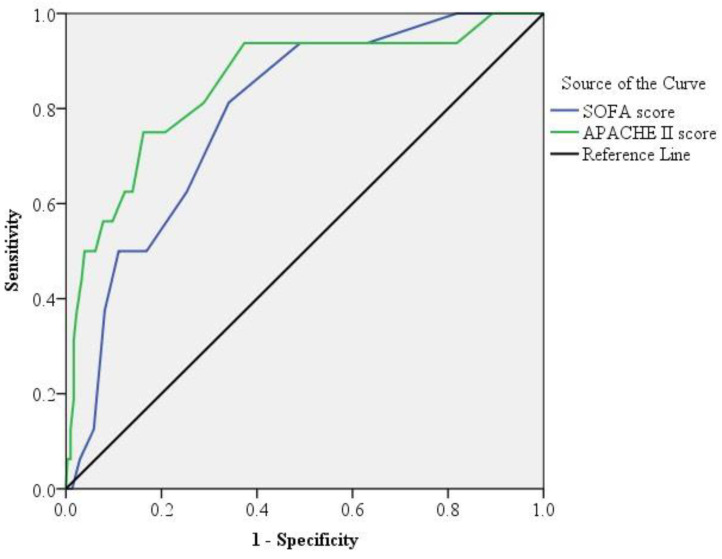
Receiver operating characteristic curve analysis. The area under the receiver operating characteristic (ROC) curve analysis for the acute physiology and chronic health evaluation II (APACHE II, green line) score was significantly higher than that for sequential organ failure assessment (SOFA, blue line) score (*p* < 0.001) in patients with pyogenic liver abscess.

Figure 2 depicts the ROC curves for evaluating the APACHE II and SOFA scores of the 324 patients with PLA. The AUC of the APACHE II score was 0.851 (±0.054; *p* < 0.0001), indicating the APACHE II score’s discriminative power for predicting the 30-day mortality. Using the Youden index, the optimal cutoff estimate for the APACHE II score for predicting 30-day mortality was 18, with a sensitivity of 75% and a specificity of 84%. Similarly, the AUC of the SOFA score was 0.787 (±0.049; *p* < 0.0001), with an optimal cutoff estimate of 6, sensitivity of 81%, and specificity of 66%.

**Figure 3:**
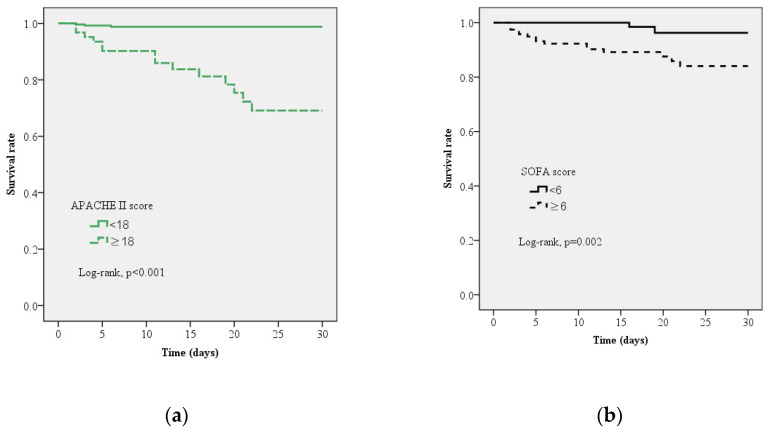
Kaplan–Meier curve estimates of the survival probability for all-cause mortality at 30-days. (**a**) A significant increase in 30-day mortality occurred with sequential organ failure assessment (SOFA) score ≥6, log-rank *p* = 0.002 (95% confidence limits, 95% CI 2.338–45.484) (**b**) A significant increase in 30-day mortality occurred with acute physiology and chronic health evaluation II (APACHE II) score ≥18, log-rank *p* < 0.001, 95% CI 5.010–61.203.

The Kaplan–Meier curves for 30-day survival in patients with PLA using APACHE II and SOFA scores are presented in Figure 3. Log-rank testing showed a high survival rate for patients with APACHE II scores <18 (95% CI: 5.01–61.20, *p* < 0.001; Figure 3a) and SOFA scores <6 (95% CI: 2.34–45.48, *p* < 0.001; Figure 3b).

## 4. Discussion

We have provided detailed clinical, imaging, and laboratory data on critical illness in patients with PLA. Our study found that the mortality risk among patients with PLA is associated with the following factors: APACHE II on admission and concomitant infections. Our study is the first report to predict mortality in patients who died and those who survived with PLA using APACHE II on admission.

In previous studies, the APACHE II and Emergency Department Sepsis scores on admission were reported to exhibit good discriminative power for predicting the mortality risk of patients with PLA [2,14,16,17]. Our finding of a high APACHE II score is consistent with previous studies, although this study provided additional information concerning its use as a predictor of mortality in patients with PLA on admission [2,16,17]. The patients who died with a SOFA ≥6 compared with patients who survived were statistically significant in our univariate analyses. In contrast to the APACHE II scoring system, the SOFA did not prove to be useful in predicting 30-day mortality in patients with PLA on admission. Our findings suggest that the APACHE II scoring system on admission could be useful for predicting mortality in patients with PLA. It may encourage clinicians to rapidly manage high-risk patients. Furthermore, patients with PLA with APACHE II score ≥18 had a 19.31-fold risk of 30-day mortality, whereas a SOFA score ≥6 on admission was predictive of a 1.96-fold risk of 30-day mortality; however, this was not statistically significant. These findings imply that the APACHE II score could be a good tool in characterizing illness severity in patients with PLA. The SOFA score has been reported in ICU patients with PLA in a previous study. However, it was not specifically used as a tool for predicting outcomes [9]. The APACHE II score considers patients’ age and chronic comorbidities, while the SOFA score does not consider these factors; this can explain the better discriminative power of APACHE II score compared with the SOFA score for patients with PLA [23].

In our cohort of ICU patients, many individuals presented with diabetes, anemia, thrombocytopenia, elevated aspartate aminotransferase and alkaline phosphatase levels, hyperbilirubinemia, and high blood urea nitrogen and creatinine. These findings are in concordance with prior research showing the association of these variables with poor prognosis [10,14,16,17]. Similarly, metastatic infections had been reported as an important risk factor for poor outcomes in patients with PLA [1]; however, it was not statistically significant (*p* = 0.051) in our multivariate analyses. This could be related to the small number of patients with metastatic infections. However, concomitant infections, such as septic shock, complicated with pneumonia, or urinary tract infection, had a 34.33-fold increased risk of 30-day mortality. The patients with PLA could develop invasive syndromes, such as bacteremia, meningitis, endophthalmitis, and/or necrotizing fasciitis, in addition to liver disease [24].

In this study, the all-case fatality rate, microbiological results, and imaging findings corroborated previous research [9,16,24,32]. The ICU patients with PLA presented more often with bacteremia, metastatic infections, and concomitant infections than the non-ICU patients. These findings are in agreement with those of previous studies and appear to reveal impaired immune responses, which could be due to the higher prevalence of diabetes observed in ICU patients in this study [16]. Finally, the patients with concomitant infections were predictive of a 34.33-fold risk of 30-day mortality in our multivariate analyses. We could focus on high-risk patients with concomitant infections.

Despite its strengths, this study had some limitations. First, this was a retrospective analysis at a single center, and complete blood testing was not performed in our cohort. Second, since data were obtained from electronic medical records and patient recall, errors may have been introduced inadvertently. Third, our study analyzed 69 PLA patients with negative culture results. Despite adjustments in our multivariate analyses model, it may have affected the choice of antimicrobial therapy and treatment outcome; an antimicrobial regimen is prescribed for such patients based only on the guidelines from the Surgical Infection Society and Infectious Diseases Society of America, without considering the SOFA score [33]. The predictive ability of APACHE in PLA was shown in this study, and while we attempted to use SOFA as an alternative predictor, it was not found to be useful. Finally, since the study setting was an Eastern Asian country, the study findings may be limited by the clinical treatment experiences of physicians in that area; therefore, the generalizability of results may be an issue. Furthermore, *K. pneumoniae* PLA has a high mortality rate and occurs at a higher incidence in tropical Southeast Asian regions, including Taiwan [24,34].

## 5. Conclusions

Our study showed that PLA has a high fatality rate in ICU patients. To improve outcomes in patients with PLA, we recommend that treatment and management strategies focus on those who are at a high-risk of death when evaluated upon admission using the APACHE II score. Future studies on the risk factors and mortality rates in treated patients with PLA are required.

## Figures and Tables

**Table 1:** Baseline characteristics and severity of the 324 patients with pyogenic liver abscess.

Characteristics	General Ward	Intensive Care Unit	*p*-Value
Number of patients	215	109	
Age, mean ± SD (years)	57.8 (15.8)	62.9 (15.7)	0.007
Sex, no. (%)			0.403
Male	148 (68.8)	70 (64.2)	
Female	67 (31.2)	39 (35.8)	
Comorbidities			
Diabetes mellitus, no. (%)	84 (39.1)	61 (56.0)	0.004
Hemoglobin A1c, no. (%) (*n* = 137)			0.244
≥10% (normal range 4.0–5.6%)	27 (35.5)	16 (26.2)	
Hypertension, no. (%)	74 (34.4)	52 (47.7)	0.020
Biliary disease, no. (%)	39 (18.1)	21 (19.3)	0.805
Chronic liver disease, no. (%)	43 (20.0)	20 (18.3)	0.723
Chronic kidney disease, no. (%)	6 (2.8)	7 (6.4)	0.116
Concomitant neoplasm, no. (%)	23 (10.7)	13 (11.9)	0.739
Alcohol consumption, no. (%)	74 (34.4)	33 (30.3)	0.454
Severity of illness on the day of hospitalization			
Duration of hospitalization, mean ± SD (days)	14.0 (7.9)	26.1 (24.5)	<0.001
SOFA, mean ± SD	4.0 (2.0)	7.3 (2.8)	<0.001
SOFA ≥6, no. (%)	43 (20.0)	75 (68.8)	<0.001
APACHE II score, mean ± SD	12.6 (4.1)	17.7 (6.6)	<0.001
APACHE II score ≥18, no. (%)	20 (9.3)	42 (38.5)	<0.001

APACHE II score, acute physiology and chronic health evaluation II score; SD, standard deviation; SOFA score, sequential organ failure assessment score.

**Table 2:** Treatment results, antimicrobial agent usage, complications, and outcomes of patients with pyogenic liver abscess.

Characteristics, no. (%)	General Ward (*n* = 215)	Intensive Care Unit (*n* = 109)	*p*-Value
Time from infection diagnosis to initiation of antibiotic use, mean ± SD (days)	1.14 (0.9)	1.27 (1.5)	0.358
Duration of parenteral antibiotics, mean ± SD (days)	14.0 (7.9)	21.7 (16.8)	<0.001
Subsequent antibiotic treatment ^a^, no. (%)	82 (38.1)	60 (55.0)	0.004
Inappropriate antibiotic therapy, no. (%)	13 (6.0)	11 (10.1)	0.189
Antibiotics used, no. (%)			0.001
Monotherapy	173 (80.5)	69 (63.3)	
Combination therapy	42 (19.5)	40 (36.7)	
Percutaneous catheter drainage, no. (%)	116 (54.0)	66 (60.6)	0.258
Clinical response, no. (%)			0.008
Response	184 (85.6)	80 (73.4)	
Non-response	31 (14.4)	29 (26.6)	
Surgical intervention, no. (%)	6 (2.8)	11 (10.1)	0.005
Complications			
Metastatic infections, no. (%) (*n* = 324)	8 (3.7)	9 (8.3)	0.032
Abscess rupture, no. (%) (*n* = 324)	2 (0.9)	3 (2.8)	0.209
Septic shock, no. (%)	25 (11.6)	12 (11.0)	0.501
Concomitant infection ^b^, no. (%) (*n* = 324)	25 (11.6)	66 (60.6)	<0.001
Mortality, no. (%)	1 (0.5)	23 (21.1)	<0.001
30-Day mortality, no. (%)			<0.001
Alive	214 (99.5)	86 (78.9)	
Death directly attributed to PLA	1 (0.5)	15 (13.8)	
Death due to complication or comorbidity	0	8 (7.3)	

SD, standard deviation; UTI, urinary tract infection; PLA, pyogenic liver abscess. ^a^ Subsequent antibiotic treatment: antibiotics were modified accordant to the microbiological culture and susceptibility results or clinical response after antibiotic administration for ≥48 h. ^b^ Concomitant infections: septic shock (*n* = 37); UTI (*n* = 18); pneumonia (*n* = 14); septic shock and pneumonia (*n* = 11); septic shock with UTI (*n* = 5); septic shock with UTI and pneumonia (*n* = 4); UTI and pneumonia (*n* = 2).

**Table 3:** Factors related to 30-day mortality in pyogenic liver abscess patients.

Variables	Univariate Analysis		Multivariable Analysis	
	OR (95% CI)	*p*-Value	aOR ^a^ (95% CI)	*p*-Value
Baseline Characteristics				
Age	1.05 (1.02–1.09)	0.001	1.04 (0.99–1.09)	0.119
Diabetes mellitus				
No	1.00		1.00	
Yes	1.50 (0.65–3.47)	0.338	0.92 (0.27–3.19)	0.893
Hypertension				
No	1.00		1.00	
Yes	2.35 (1.01–5.47)	0.047	2.56 (0.71–9.27)	0.153
SOFA				
<6	1.00		1.00	
≥6	14.65 (4.27–50.30)	<0.001	1.96 (0.38–10.07)	0.418
APACHE II score				
<18	1.00		1.00	
≥18	30.71 (10.00–94.32)	<0.001	19.31 (4.77–78.22)	<0.001
Laboratory data and Complications				
Blood samples results				
No growth	1.00		1.00	
Growth of blood samples	1.35 (0.57–3.17)	0.498	0.47 (0.07–3.36)	0.453
Metastatic infections				
No	1.00		1.00	
Yes	2.92 (0.78–10.96)	0.113	9.74 (0.91–104.22)	0.060
Concomitant infections				
No	1.00		1.00	
Yes	36.83 (8.45–160.53)	<0.001	34.33 (5.60–210.55)	<0.001

OR, adjusted odds ratio; APACHE II score, acute physiology and chronic health evaluation II score; CI, confidence interval; OR, odds ratio; SOFA score, sequential organ failure assessment score. ^a^ Adjusted clinically significant variables on admission including age, diabetes mellitus, hypertension, SOFA score, APACHE II score, pathogens isolated from blood sample, metastatic infections, and concomitant infections.

## Data Availability

The datasets generated during and/or analyzed during the current study are available from the corresponding author (Yuan-Ti Lee) on reasonable request.

## Supplementary Materials

The following are available online at https://www.mdpi.com/article/10.3390/jcm10122644/s1, Table S1: Clinical Manifestations and Laboratory findings of the 324 Patients with Pyogenic Liver Abscess; Table S2: Microbiology results, Imaging findings, and Complications of the 324 Patients with Pyogenic Liver Abscess.
