# Supplementary material for: Utility of Acute Physiology and Chronic Health Evaluation (APACHE II) in Predicting Mortality in Patients with Pyogenic Liver Abscess: A Retrospective Study
Source: J Clin Med. 2021 Jun 16;10(12):2644. doi: 10.3390/jcm10122644 (PMC8235429; doi:10.3390/jcm10122644)
Supplement: Supplementary file 1 [file jcm-10-02644-s001.zip › jcm-1206346-supplementary.pdf]

Table S1. Clinical Manifestations and Laboratory findings of the 324 Patients with Pyogenic Liver Abscess.

Characteristics	General Ward (n = 215)	Intensive Care Unit (n = 109)	*p-value*
Symptoms when hospitalization			
Chills, no. (%)	153 (71.2)	64 (58.7)	0.024
Weight loss, no. (%)	17 (7.9)	13 (11.9)	0.238
Malaise, no. (%)	206 (95.8)	107 (98.2)	0.270
Cough, no. (%)	53 (24.7)	21 (19.3)	0.275
Chest pain or discomfort, no. (%)	22 (10.2)	21 (19.3)	0.024
Shortness of breath, no. (%)	27 (12.6)	24 (22.0)	0.027
Abdominal pain, no. (%)	107 (49.8)	53 (48.6)	0.846
Poor appetite, no. (%)	79 (36.7)	42 (38.5)	0.753
Nausea or vomiting, no. (%)	54 (25.1)	29 (26.6)	0.772
Diarrhea, no. (%)	26 (12.1)	10 (9.2)	0.430
Duration of the symptoms before hospitalization (days), no. (%)			0.055
Days ≤3	92 (42.8)	61 (56.0)	
3<Days≤7	78 (36.3)	34 (31.2)	
Days>7	45 (20.9)	14 (12.8)	
Signs when hospitalization			
Systolic blood pressure, mean±SD (mmHg)	117.9 (18.6)	108.1 (25.9)	0.001
Fever >37.8° at hospitalization °C, no. (%)	194 (90.2)	88 (80.7)	0.016*
Fever >38.3°C, no. (%)	76 (35.3)	29 (26.6)	0.112
Mental confusion, no. (%)	6 (2.8)	20 (18.3)	<0.001
Abnormal breathing sound, no. (%)	23 (10.7)	28 (25.7)	<0.001
Jaundice, no. (%)	23 (10.7)	15 (13.8)	0.418
Right upper quadrant tenderness, no. (%)	101 (47.0)	39 (35.8)	0.055
Murphy's sign, no. (%)	13 (6.0)	5 (4.6)	0.588
Ascites, no. (%)	13 (6.0)	18 (16.5)	0.002
Laboratory			
White blood cell count (4000–11,000/μL), no. (%) (n = 324)			0.432
<10,000	60 (27.9)	35 (32.1)	
≥10,000	155 (72.1)	74 (67.9)	
Hemoglobin (male 13–17 g/dL; female 12–16 g/dL), no. (%) (n = 309)			<0.001
<10	14 (6.6)	21 (21.4)	
≥10	197 (93.4)	77 (78.6)	
Platelets (150,000–400,000/μL), no. (%) (n = 324)			<0.001
<100,000	19 (8.8)	35 (32.1)	
≥100,000	196 (91.2)	74 (67.9)	
Hs-CRP (<0.4 mg/dL), no. (%) (n = 317)			0.095
<10	52 (24.9)	18 (16.7)	
≥10	157 (75.1)	90 (83.3)	
Alanine aminotransferase (3~37 IU/L), no. (%) (n = 310)			0.068
<37	90 (44.3)	36 (33.6)	
≥37	113 (55.7)	71 (66.4)	
Aspartate aminotransferase (15–41 IU/L), no. (%) (n = 272)			<0.001
<41	104 (60.5)	36 (36.0)	
≥41	68 (39.5)	64 (64.0)	
Alkaline phosphatase (43–122 IU/L), no. (%) (n = 249)			0.017
<122	89 (56.3)	37 (40.7)	

≥122	69 (43.7)	54 (59.3)	
Total bilirubin (0.2–1.2 mg/dL), no. (%) (*n* = 272)			0.039
<1.3	98 (57.0)	44 (44.0)	
≥1.3	74 (43.0)	56 (56.0)	
γ- glutamyl transferase (7–50 IU/L), no. (%) (*n* = 237)			0.688
<50	57 (36.8)	28 (34.1)	
≥50	98 (63.2)	54 (65.9)	
Fasting glucose (60–100 mg/dl), no. (%) (*n* = 283)			0.136
<126	55 (30.6)	23 (22.3)	
≥126	125 (69.4)	80 (77.7)	
Blood urea nitrogen (8–20 mg/dL), no. (%) (*n* = 273)			<0.001
<20	115 (67.3)	36 (35.3)	
≥20	56 (32.7)	66 (64.7)	
Creatinine (0.5~1.3 mg/dL), no. (%) (*n* = 324)			<0.001
<1.3	166 (77.2)	50 (45.9)	
≥1.3	49 (22.8)	59 (54.1)	

hs-CRP, high-sensitivity C-reactive protein; SD, standard deviation.

Table S2. Microbiology results, Imaging findings, and Complications of the 324 Patients with Pyogenic Liver Abscess.

**Characteristics, no. (%)**	**General Ward (*n* = 215)**	**Intensive Care Unit (*n* = 109)**	***p-value***
Microbiologic studies			
Classification of samples			0.001
No growth	54 (25.1)	15 (13.8)	
growth of blood and abscess samples	60 (27.9)	43 (39.4)	
growth of abscess samples	58 (27.0)	16 (14.7)	
growth of blood samples	43 (20.0)	35 (32.1)	
Blood samples			<0.001
No growth	112 (52.1)	31 (28.4)	
K. pneumoniae[a]	78 (36.3)	56 (51.4)	
E. coli	11 (5.1)	8 (7.3)	
Other GNB	13 (6.0)	8 (7.3)	
GPC	1 (0.5)	6 (5.6)	
Abscess samples[b]			0.347
No growth	97 (45.1)	50 (45.9)	
K. pneumoniae[a]	94 (43.7)	42 (38.5)	
E. coli	9 (4.2)	5 (4.6)	
Other GNB	11 (5.1)	7 (6.4)	
GPC	4 (1.9)	5 (4.6)	
Susceptibility testing of blood samples			0.027
Non-multidrug-resistant	180 (83.7)	80 (73.4)	
Multidrug-resistant	35 (16.3)	29 (26.6)	
Susceptibility testing of abscess samples			0.250
Non- multidrug-resistant	170 (79.1)	80 (73.4)	
Multidrug-resistant	45 (20.9)	29 (26.6)	
Susceptibility testing of blood and abscess samples		0.066	
Non- multidrug-resistant	152 (70.7)	66 (60.6)	
Multidrug-resistant	63 (29.3)	43 (39.4)	
Imaging studies			
Abdominal ultrasound abscess sizes in area (*n* = 280)			0.423
<5 cm^2	32 (16.9)	19 (20.9)	
≥5cm^2	157 (83.1)	72 (79.1)	
Abdominal ultrasound abscess numbers (*n* = 280)			0.068

1	146 (77.2)	61 (67.0)	
≥2	43 (22.8)	30 (33.0)	
Abdominal CT abscess sizes in area (*n* = 304)			0.473
<5 cm^2	29 (14.5)	12 (11.5)	
≥5cm^2	171 (85.5)	92 (88.5)	
Abdominal CT abscess numbers (*n* = 304)			0.168
1	146 (73.0)	68 (65.4)	
≥2	54 (27.0)	36 (34.6)	

CT, Computerized Tomography; GNB, Gram-negative bacteria; GPC, Gram-positive cocci; *E. coli*, *Escherichia coli*; *K. pneumoniae*, *Klebsiella pneumoniae*; [a]A total of 182 isolates of *K. pneumoniae* was isolated, including 134 isolates from blood samples, 132 isolates from abscess samples, and 84 isolates from both blood and abscess samples. [b]A total of 255 isolates was isolated, including 78 isolates from blood samples, 74 isolates from abscess samples, and 103 species from both blood and abscess samples.
